# Shell-Isolated Nanoparticle-Enhanced Raman Spectroscopy

**DOI:** 10.3389/fchem.2019.00410

**Published:** 2019-06-04

**Authors:** Jan Krajczewski, Andrzej Kudelski

**Affiliations:** Faculty of Chemistry, University of Warsaw, Warsaw, Poland

**Keywords:** shell-isolated nanoparticle-enhanced Raman spectroscopy, surface-enhanced Raman spectroscopy, core-shell nanoparticles, SHINERS, SERS

## Abstract

In 2010, Tian et al. reported the development of a new, relatively sensitive method of the chemical analysis of various surfaces, including buried interfaces (for example the surfaces of solid samples in a high-pressure gas or a liquid), which makes it possible to analyze various biological samples *in situ*. They called their method shell-isolated nanoparticle-enhanced Raman spectroscopy (SHINERS). SHINERS spectroscopy is a type of surface-enhanced Raman spectroscopy (SERS) in which an increase in the efficiency of the Raman scattering is induced by plasmonic nanoparticles acting as electromagnetic resonators that locally significantly enhance the electric field of the incident electromagnetic radiation. In the case of SHINERS measurements, the plasmonic nanoparticles are covered by a very thin transparent protective layer (formed, for example, from various oxides such as SiO_2_, MnO_2_, TiO_2_, or organic polymers) that does not significantly damp surface electromagnetic enhancement, but does separate the nanoparticles from direct contact with the probed material and keeps them from agglomerating. Preventing direct contact between the metal plasmonic structures and the analyzed samples is especially important when biological samples are investigated, because direct interaction between the metal nanoparticles and various biological molecules (e.g., peptides) may lead to a change in the structure of those biomolecules. In this mini-review, the state of the art of SHINERS spectroscopy is briefly described.

## Introduction

**Figure 1:**
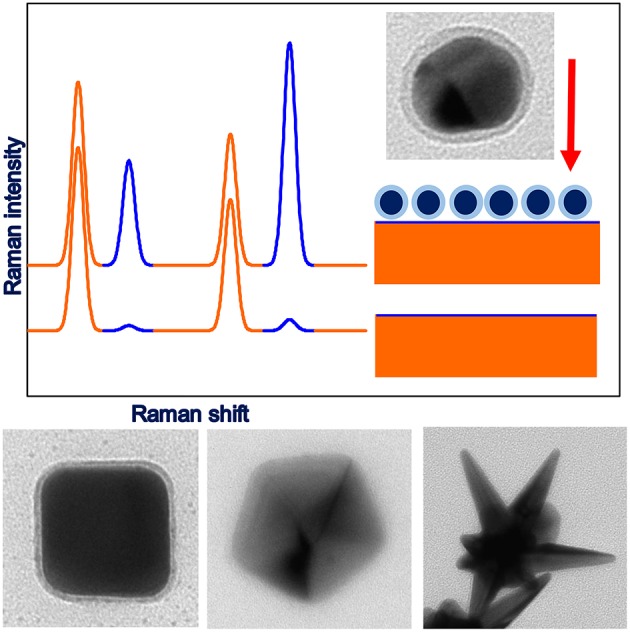
Upper part: illustration of the principles of SHINERS measurements, lower part: example TEM images of anisotropic SHINERS nanoresonators (cubic-Ag@MnO_2_, decahedral-Ag@SiO_2_, star-Au@SiO_2_).

When nanoparticles formed from metals with a negative real and small positive imaginary dielectric constant (for example, nanoparticles of gold or silver) interact with electromagnetic radiation, a collective oscillation of surface conduction electrons called surface plasmons is induced (Aroca, 2006). The excitation of surface plasmons significantly affects the intensity of the electric field in the proximity of the illuminated plasmonic nanoparticles—a very large enhancement of the electromagnetic field may be induced in some places in the proximity of the illuminated plasmonic nanostructures (Wei et al., 2018). This electric field enhancement leads to a significant increase in the efficiency of a number of optical processes for molecules in the proximity of the plasmonic nanostructures, such as: fluorescence (Touahir et al., 2010; Wang et al., 2012), phosphorescence (Meng et al., 2018), second harmonic generation (Brolo et al., 2002), Raman scattering (Brolo et al., 2002; Wang et al., 2013; Zhang et al., 2014), Raman optical activity (Osinska et al., 2010), hyper-Raman scattering (Hulteen et al., 2006), coherent anti-Stokes Raman scattering (Brolo et al., 2002), and infrared absorption (Imae and Torii, 2000). From the practical point of view, what is most important is the enhancement of the efficiency of Raman scattering generation induced by the plasmonic systems–this effect is called surface-enhanced Raman scattering (SERS). A very interesting kind of SERS spectroscopy developed in 2010 by Tian et al. is known as shell-isolated nanoparticle-enhanced Raman spectroscopy—SHINERS (Li et al., 2010). In this method, plasmonic nanoparticles are covered with a very thin protective layer, and the core-shell structures obtained are deposited on the surface under study. The plasmonic cores of the deposited nanoparticles induce a large local enhancement in the intensity of the electric field, which, as in standard SERS measurements, leads to a significant increase in the efficiency of the Raman signal generation. The deposited ultrathin protective layer only slightly dampers the electromagnetic enhancement, but also prevents the nanoparticles from coming into direct contact with the probed material, and from agglomerating. Preventing direct contact between the metallic plasmonic resonators and the surfaces being investigated is especially important when biological samples are analyzed, because direct interaction between the metal plasmonic structures and various biological molecules (e.g., peptides) may lead to a change in the structure of the biomolecules. The SHINERS spectroscopy developed by Tian et al. seems be a very useful tool for analyzing the surfaces of various materials, even in *in situ* conditions, which is a big advantage both economically and scientifically. For example, by depositing SHINERS nanoresonators on living cells, one can record *in situ* the Raman spectrum dominated by the contribution from the species present in the outermost part of the cells of various organisms (Li et al., 2010; Kołataj et al., 2017). The principles of SHINERS measurements are presented in Figure 1. In this mini-review, we briefly present current advances in the synthesis of SHINERS nanoresonators (both plasmonic cores and protective layers), and new applications of SHINERS spectroscopy.

## Synthesis of Plasmonic Cores for Shiners Nanoresonators

The first SHINERS experiments were carried out using, as plasmonic cores, spherical gold nanoparticles synthesized by the standard citrate method (Li et al., 2010, 2011a,b; Butcher et al., 2012). In 2012, Tian et al. (Uzayisenga et al., 2012) and Kudelski and Wojtysiak (2012) carried out the first SHINERS measurements using silver rather than gold nanoresonators, and showed that, in some cases, the use of silver plasmonic cores allows for a significant increase in the sensitivity of the SHINERS measurements. The silver plasmonic cores for the first SHINERS measurements were also spherical and were synthesized using the standard citrate method (Kudelski and Wojtysiak, 2012; Uzayisenga et al., 2012). Recently, also bimetallic plasmonic core-shell structures have been applied as cores of SHINERS nanoresonators (Lin et al., 2015).

As has been shown in many theoretical simulations, the highest field enhancement is induced in sharp structures on the surface of the illuminated plasmonic nanoparticles. Therefore, it is well-known that an effective method of increasing the efficiency of the enhancement of the intensity of the electric field in the plasmonic nanoparticles is to form on their surfaces various sharp structures, such as apexes and edges (Kottmann et al., 2001; Kudelski, 2009). Moreover, for anisotropic nanoparticles, the wavenumber of the radiation that induces the surface plasmon resonance can be changed in a significantly broader frequency range than for spherical nanostructures. Due to higher activity in the SERS measurements, and because it is easier to change the condition of the surface plasmon resonance, anisotropic plasmonic nanostructures having the shape of a cube, rod, dipyramide, decahedral, or star have been tested as plasmonic cores of SHINERS nanoresonators. As expected, when anisotropic plasmonic nanoparticles containing many sharp apexes and edges are used as SHINERS nanoresonators, the intensity of the measured Raman signal is significantly greater than in the case of experiments carried out using spherical plasmonic nanoparticles having roughly the same size and produced from the same amount of plasmonic material. For example, in the case of nanoparticles having the shape of a decahedron or dipyramid, the increase in SERS activity is equal to about one order of magnitude (Kołataj et al., 2017, 2018).

Anisotropic plasmonic nanostructures can be often synthesized using relatively simple chemical methods. For example, gold nanorods and dipyramids can be obtained using what is known as seed mediated growth synthesis (Quyan et al., 2014). In this process, anisotropic nanoparticles are formed as a result of the growth (achieved by a reduction of the metal precursor, for example HAuCl_4_, by a weak reducing agent like ascorbic acid) of initially spherical seeds in solutions containing various surfactants. Silver decahedrons are usually obtained by photochemical synthesis (Kołataj et al., 2018). Anisotropic plasmonic nanoparticles can be also synthetized using various compounds that are selectively adsorbed on various crystalline facets. For example, a reduction of silver ions in hot dimethylformamide in the presence of polyvinylpyrrolidone leads to the formation of silver nanodecahedrons (Gao et al., 2006), while a reduction of silver ions in hot ethylene glycol in the presence of sulfide ions leads to the formation of silver nanocubes (Siekkinen et al., 2006)—all these kinds of nanoparticles have been tested as plasmonic cores for SHINERS nanoresonators (Quyan et al., 2014; Kołataj et al., 2018).

Another interesting kind of plasmonic cores for SHINERS nanoresonators are hollow nanostructures, which exhibit in many cases plasmonic properties superior to analogous solid nanoparticles (Sun et al., 2003; Huschka et al., 2011). For example, for hollow spherical gold nanostructures the position of the plasmonic band can be changed (by changing the diameter of the nanoparticle and the thickness of the shell) in a significantly broader wavelength range than for solid spherical gold nanoparticles (Sun et al., 2003; Huschka et al., 2011). The significant achievable red-shift of the plasmon band for hollow gold nanoparticles facilitates various biomedical applications of such nanostructures, because it makes it possible to tune the position of the plasmonic band to the transparent window of typical biological tissues (800–1,200 nm) (Hirsch et al., 2003; Prodan et al., 2003). Therefore, SHINERS nanoresonators containing hollow plasmonic cores are especially useful for experiments in which red or infrared radiation must be used.

In 2015, Abdulrahman et al. (2015) reported the first example of SHINERS measurements using hollow silver and hollow gold nanoparticles as plasmonic cores of SHINERS nanoresonators. Similar to SHINERS nanoresonators containing anisotropic cores, SHINERS nanoresonators containing hollow plasmonic cores are in many cases significantly more efficient than solid nanostructures of a similar size—especially when it is possible to significantly change the frequency of the surface plasmon resonance for the synthesized systems (Abdulrahman et al., 2015).

## Formation of the Protective Layer

As mentioned in the introduction, in order to obtain SHINERS nanoresonators, the plasmonic cores have to be covered with a dielectric protective layer. Protective layers are typically formed from various oxides such as SiO_2_, MnO_2_, TiO_2_, or ZrO_2_. However, in some cases, organic polymers or carbon shells are also used. Nowadays, the cores of SHINERS nanoresonators are often covered with an silica layer, which is usually formed by the decomposition of Na_2_SiO_3_ or tetraethyl orthosilicate. Deposition of a nanometric silica layer by the decomposition of Na_2_SiO_3_ is usually carried out using the procedure proposed by Mulvaney et al. (2003). Briefly speaking, to a sol of metal nanoparticles (Ag or Au), an Na_2_SiO_3_ solution acidified with HCl is added, and the reaction mixture is kept for a relatively long time (even up to 6 days) under stirring. In the case of the deposition of silica on highly anisotropic nanoparticles, their surfaces are often protected by a layer formed from alkanethiols, for example, from 16-mercaptohexadecanoic acid (Mulvaney et al., 2003).

A silica layer can be also formed by the catalyzed by ammonia or amines (like dimethylamine) decomposition of tetraethyl orthosilicate. This reaction is carried out in organic solvents such as isopropanol (Krajczewski et al., 2018b) or ethanol (Kobayashi et al., 2005). The thickness of the silica shells formed depends on the initial concentration of the tetraethyl orthosilicate added. An important advantage of this method is the short time required to deposit even a thick SiO_2_ layer—usually only *ca*. 15 min.

A serious drawback of silica protective layers is their low durability in some solutions, for example, solutions having a high or low pH. Therefore, for SHINERS investigations in alkali media, Tian et al. suggested using Au@MnO_2_ nanoresonators (Liu et al., 2012). Layers of MnO_2_ on gold cores have been deposited by a reduction of KMnO_4_ by K_2_C_2_O_4_ carried out in the presence of Au nanoparticles in a solution with a pH of 9.5 (Liu et al., 2012). The thickness of the MnO_2_ layer formed can be controlled by changing the concentration of KMnO_4_. To deposit an MnO_2_ layer on silver cores, the above procedure has to be slightly modified—silver cores dissolve themselves in such a reaction mixture (Abdulrahman et al., 2016). In order to deposit MnO_2_ layer on Ag cores, the reduction of KMnO_4_ by K_2_C_2_O_4_ has to be carried out at a higher pH (optimally at pH equal to *ca*. 12) (Abdulrahman et al., 2016).

SHINERS nanoresonators protected by ZrO_2_ layers are significantly more stable in acidic and alkaline solutions than nanoresonators protected by SiO_2_ shells. Moreover, since zirconia has an exceptionally large refractive index, the deposition of a ZrO_2_ layer significantly shifts the frequency of the plasmon resonance. A zirconia layer is usually deposited by the decomposition of zirconium (IV) isopropoxide (Krajczewski et al., 2018a).

SHINERS nanoresonators can be also protected by carbon or the polymer layers. To form carbon shells, Yang et al. deposited p-mercaptobenzoic acid on the surface of silver nanoparticles and then carbonized the deposited organic layer in a concentrated solution of sulphuric acid. TEM analysis of the nanostructures obtained showed that, after this procedure, the silver cores are covered by a carbon layer with a thickness of about 2 nm (Yang et al., 2011). SHINERS nanoresonators protected by a layer of polydopamine have been synthesized by Ye et al. (2017). Polydopamine layers on gold cores were formed by the self-polymerization of dopamine, and the thickness of the layer can be controlled by changing the dopamine concentration.

An important problem in the synthesis of SHINERS nanoresonators is how to control the thickness of the protective layer. If the layer is too thin, pin-holes are more likely to appear. However, increasing the thickness of the protective layer deposited on the SHINERS nanoresonators leads to a decrease in their SERS activity [the achievable SERS enhancement decreases as a function of r^−10^ with increasing distance from the plasmonic nanoparticle (Stiles et al., 2008)]. More details on the dependence of the efficiency of SHINERS nanoresonators in enhancing in the efficiency of Raman scattering on the thickness of a deposited layer is given by Stiles et al. (2008).

## Example applications of SHINERS spectroscopy

SHINERS spectroscopy has been used to detect and determine the concentration of many various chemical compounds or biological objects. One of the first systems analyzed using SHINERS spectroscopy was the skin of an orange fruit contaminated with methyl parathion (Li et al., 2010). The SHINERS detection of methyl parathion (an efficient insecticide) is based on the observation of the appearance of its characteristic Raman band at 1,350 cm^−1^. The first SHINERS detection of methyl parathion was carried out using Au@SiO_2_ nanoparticles; later, another nanoresonators were also used, for example Ag@SiO_2_ (Kudelski and Wojtysiak, 2012), hollow–Ag@SiO_2_ (Abdulrahman et al., 2015), and Ag@MnO_2_ nanoparticles (Abdulrahman et al., 2016). Another pesticide detected using SHINERS spectroscopy was thiram (Kołataj et al., 2018). The limit of detection of thiram on a tomato skin using SHINERS spectroscopy was estimated as 0.9 ng/cm^2^ (Kołataj et al., 2018).

Other important compounds that can be detected using SHINERS spectroscopy are nitrites. Nitrite ions (${\text{NO}}_{2}^{-}$) pose a serious hazard to human health (Ralt, 2009) and the environment (Chung et al., 2004), and therefore, the US Environmental Protection Agency established a maximum contaminant level allowed for nitrite ions in drinking water (equal to 1 mg/L) (Zhang et al., 2013). Nitrite ions do not produce strong Raman spectra. Also, the addition of gold nanoparticles does not lead nitrite ions producing strong SERS spectra. However, it has been shown that nitrite ions can be detected by using them in the diazotization of p–nitroaniline in acid media. Diazotization leads to a coupling reaction and to the formation of azo dye, which has a very large cross-section for Raman scattering. The azo dye formed exhibits strong SHINERS spectra, meaning that nitrite ions can be detected indirectly using the SHINERS technique on the basis of the intensity of the Raman signal of the azo dye (Zhang et al., 2013). The limit of detection was estimated as 0.07 mg/L (Zhang et al., 2013). The method described has been successfully applied for the analysis of real samples of drinking water, tap water, and mineral water. Importantly, this method exhibits high selectivity against nitrite ions.

SHINERS measurements could be applied to detect illegal food additives, like melamine (Yang et al., 2017). Application of Ag@SiO_2_ allows to detect melamine in milk samples with good reproducibility, ultrahigh sensitivity, and high stability in a wide concentration range (0.1–5 ppm).

**Figure 2:**
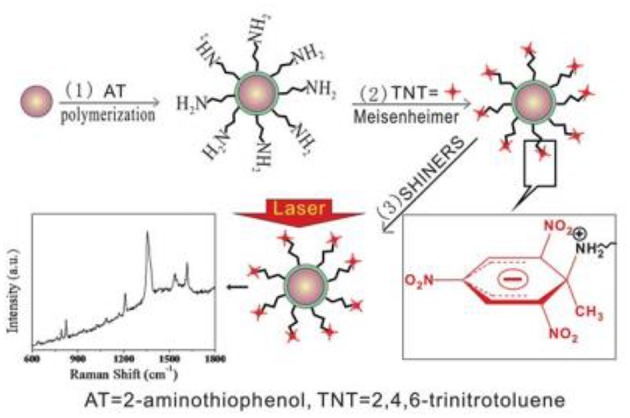
General scheme of the formation of Meisenheimer complexes between molecules of trinitrotoluen and Au@PAT nanoparticles (Qian et al., 2012). The formation of Meiseinheimer complexes makes it possible to detect trinitrotoluen molecules using SHINERS spectroscopy. Reproduced with permission from Royal Society of Chemistry, 2012.

An interesting class of SHINERS nanoresonators is nanoresonators containing organic shells. For example, gold cores coated by poly(2-aminothiophenol) (PAT) have been used to detect trinitrotoluen (Qian et al., 2012). Due to the strong electron-withdrawing effect of the nitro group, trinitrotoluen forms Meisenheimer complexes with the amino groups which come from PAT deposited on gold nanoparticles (see Figure 2). The formation of Meisenheimer complexes is observed even when traces of trinitrotoluen are present in the gas phase. The detection of trinitrotoluen is based on measuring the intensity of the characteristic Raman bands of the complex formed (Qian et al., 2012). The sensor described exhibits high selectivity because the presence in the analyzed sample of dinitrotoluene, nitrophenol, nitrobenzene, or toluene does not generate any significant Raman signal.

Gold SHINERS nanoresonators covered by polydopamine have been used for the label-free and quantitative detection of benzotriazole, an important corrosion inhibitor, by utilizing a presumed π-π stacking interaction (Ye et al., 2017). A broad linear range from 10^−4^ to 10^−8^ M was achieved with a limit of detection of 1 nM. The limit of detection of benzotriazole using SHINERS spectroscopy is about 200 times lower than the maximum allowable level of benzotriazole in water, and is also significantly lower than that of some modern methods such as gas chromatography coupled with mass spectrometry, liquid chromatography, and fluorescence (Ye et al., 2017).

SHINERS measurements could be applied to monitored some catalytic reactions (Hartman et al., 2018; Wang et al., 2019), for example, oxidation of methanol and formic acid (Wang et al., 2018). Also the mechanism of CO oxidation could be investigated using SHINERS spectroscopy (Zhang et al., 2017a). In a case of reduction of p–nitrothiophenol to p–aminothiophenol by sodium borohydride carried out in a liquid phase SHINERS nanoparticles demonstrate the bifunctionality as SERS nanoresonators and nanocatalyst (Zhang et al., 2017b). SHINERS is also one of the novel and promising techniques for electrochemical surface science, which can be relatively easy carried out in aqueous solutions (Xu et al., 2018).

Another promising field of application of SHINERS nanomaterials is the detection of various biochemical compounds and the analysis of biological samples. For example, it has been reported that, when SHINERS nanoresonators are deposited on various cells, it is possible to differentiate, on the basis of the measured SHINERS spectra, between normal and pathologically-changed cancer cells (Liang et al., 2014; Zheng et al., 2014). Kast et al. (2008) also showed that pathologically-changed tissues exhibit slightly different SHINERS spectra, which makes it possible to identify various types of cancer.

Due to their non-toxicity and high biocompatibility SHINERS nanoresonators could be sometimes applied for detection of biomolecules *in vivo*. For example, Au@SiO_2_ nanoparticles have been used to investigate the silica nano-biointeraction inside eukaryotic cells *in situ* (Drescher et al., 2014). Ag@SiO_2_ nanoparticles could selectively enhance Raman signal of cytochrome c, while, due to the very weak interaction with the target molecule, the important properties of this protein are not affected (Sivanesan et al., 2012). Also secondary structures of proteins could be investigated by SHINERS method (Wang et al., 2012). Therefore, SHINERS spectroscopy is very promising tool for non-invasive optical analysis of biomolecular processes.

## Summary

In this mini-review, we briefly presented recent advances in the synthesis of SHINERS nanoresonators (both plasmonic cores and protective layers) and some typical applications of SHINERS spectroscopy in chemical and biochemical analysis. We hope that these examples convince readers that SHINERS spectroscopy is a sensitive tool useful in the chemical analysis of various surfaces, including surfaces of biological objects in *in situ* conditions.

## Author Contributions

All authors listed have made a substantial, direct and intellectual contribution to the work, and approved it for publication.

### Conflict of Interest Statement

The authors declare that the research was conducted in the absence of any commercial or financial relationships that could be construed as a potential conflict of interest.
